# Annotations

**Published:** 1902-11-29

**Authors:** 


### ANNOTATIONS.

### Less than Justice.

The case at the Old Bailey last week, when Mrs. Penruddocke was convicted after a two days' trial of cruelty to her little daughter, is one which reflects little credit upon our judicial system. Here was a woman of education, holding a high social position, possessed of large means and in the enjoyment of every comfort which money can supply, systematically tormenting her youngest child, as the jury found, almost from its birth. The fact, which the evidence in our judgment did not bear out fully, that the jury did not find the mother guilty of having done grievous bodily harm resulting in permanent mischief to the child, was no justification for the Judge treating Mrs. Penruddocke so differently from persons in a humbler position who have been convicted of cruelty to their children. She should certainly have been consigned to prison, and to impose a paltry fine of £50, was to do less than justice. The medical aspects of the case aggravate the cruelty of the mother and make it the more reprehensible that she should have received such tender consideration at the hands of the court officials and the judge, when in reality the verdict of the jury proves her to have been brutal, unnatural and a criminal liable to imprisonment. We always feel that the husband ought to be associated with the wife in these cases. Here, as the jury properly found, the husband was greatly to blame and we would like to see our laws so amended as to secure that in all cases relating to the sexes both the man and the woman should be put in the dock and tried together. This lenient and unjust treatment of Mrs. Penruddocke is calculated to bring contempt upon our judges and the law, and for this reason we hope steps may be taken in the direction of securing a revision of the judgment, for otherwise permanent injury may be done.

### "The Writing on the Wall."

In his annual report which has just been presented, Dr. Williams, Medical Officer of the Port of London, refers to the recent spread of cholera in the East as the "writing on the wall," which indicates only too probably that next summer the Port Sanitary Authority and their officers will have to face the responsible work of preventing the introduction of that disease into London. We do not think that there is any reason for alarm on the subject, but undoubtedly Dr. Williams is in the right in drawing attention to the matter. Not only from the East, as represented by Egypt and the neighbouring countries, but from the very Far East, from China, Japan, and Manilla, come reports of a very wide extension of cholera during recent months. In Egypt the disease is steadily diminishing in intensity, but this year's outbreak has been a severe one, the returns having run up at one time to as many as 1,500 deaths per day. During the season of the pilgrimage the deaths from cholera in the Hedjaz must have amounted to over 3,000, besides a good many which took place on the Yemen coast. More lately a considerable outbreak has occurred in Syria, and is still in progress. A large number of deaths have occurred in Palestine, the returns showing that the disease is widespread and has taken a considerable hold of the country. Turning now to the Far East the reports show a remarkable recrudescence of the disease. It is estimated that 75,000 cases of cholera have occurred in the Philippine Islands since last March, with a mortality of 75 per cent. In Japan recent advices show that there have been 4,329 cases and 1,650 deaths, while in Hong Kong there have been 459 cases and 396 deaths since the beginning of the outbreak. This colony, however, is now declared free from cholera. The disease is said to have been epidemic at Nanking, where 40,000 deaths are reported, while Shoo-Yanghsien reports 3,000 cases a day. Evidently cholera is once more upon the warpath, and, as is its usual course, is levying toll upon ill-drained, badly-watered, insanitary countries. Experience, however, has shown that for places which are well drained and supplied with good water cholera has but few terrors, especially when due care is taken, as we believe it will be taken by our sanitary authorities, to follow up and isolate all suspicious imported cases.

### Convalescent Homes in London Suburbs.

The London County Council has instructed the Parks and Open Spaces Committee to consider and report what houses and mansions situated in the parks and open spaces under the control of the Council can be leased for the purpose of being used as convalescent homes for children of the poorer class in London. It is the intention, we believe, of the members of the Council who are moving in this matter, to give preference to those children who have been under treatment at the various non-infectious hospitals. It appears from inquiries we have made that at Clissold Park and Avery Hill there are large houses, the whole or a greater portion of which have been empty for a long time, and at Golder's Hill a house has been occupied as a convalescent home during the past two years for the reception of soldiers. We are of course aware that this proposal of the London County Council has met with some measure of opposition, but, if it can be shown that the houses in question are suitable for the purpose, that they are not needed for other public requirements, and that the reception of convalescent cases into these buildings will in no way interfere with the enjoyment of the parks and open spaces by the people, for whose advantage they have been established and are at present maintained, we believe that public opinion will approve the scheme. It cannot be doubted that a great amount of good might be done to hundreds and possibly thousands of London children by utilising the present vacant buildings as convalescent homes. The situation of these buildings renders them better adapted on economical grounds for convalescent purposes in the case of the poorer children than those which are situated miles away in the country. We shall watch the development of this new departure on the part of the County Council with interest. It indicates a recognition of the claims and needs of the poorest citizens on the part of the Council which we welcome in these material days. Sir William Collins is always labouring to make London government in these matters an example to other cities, a laudable endeavour in which he is loyally supported by his colleagues on the Council.

